# Integrating education-based interventions and machine learning for stunting prevention: A case study in East Lombok, Indonesia

**DOI:** 10.1016/j.dialog.2025.100264

**Published:** 2025-12-06

**Authors:** Mhd. Lailan Arqam, Asno Azzawagama Firdaus, Ahmad Muslih Atmojo, Ginanjar Zukhruf Saputri, Furizal, Palahuddin, Retno Sirnopati

**Affiliations:** a Department of Islamic Education, Universitas Ahmad Dahlan, Yogyakarta 55166, Indonesia; b Department of Computer Science, Universitas Qamarul Huda Badaruddin Bagu, Central Lombok 83562, Indonesia; c Department of Pharmacy, Universitas Ahmad Dahlan, Yogyakarta 55166, Indonesia; d Department of Informatics Engineering, Universitas Islam Riau, Pekanbaru 28284, Indonesia; e Department of Public Administration Science, Universitas Muhammadiyah Mataram, Mataram 83115, Indonesia; f Department of Islamic Education, IAI Qamarul Huda, Central Lombok 83562, Indonesia

**Keywords:** Machine learning, East Lombok, Health, Stunting

## Abstract

One of the regions in Indonesia that has the highest prevalence of stunting cases is West Nusa Tenggara, with a percentage of cases almost reaching 12.7 %, even though this province is a priority target for stunting reduction by 2022. Specifically in the East Lombok region, this study took this location point because of the high number of stunting cases in West Nusa Tenggara. Puskesmas Denggen was the target of the study, covering six working areas namely Denggen, East Dengen, Majidi, Rakam, Sekarteja, and Pancor, with a total of 3416 under-five data. The data were obtained through two measurements: the initial in February 2024 and the final in August 2024. This research integrates a multidisciplinary approach, encompassing health and nutrition science, psychology, education, and religion, to create comprehensive interventions for stunting prevention and employs machine learning models to predict future cases. The interventions include Motivation, Hygiene, Nutrition, Mental Health, and Infant Health, which are designed to cover all the essential needs of children in the growth and development process. The results of the six villages measured showed that significant changes in data were obtained in Denggen Village when compared before and after the intervention. The results of measuring the effectiveness of the anti-stunting educational interventions were also found to be effective across the five key aspects, with several showing dominant and statistically significant improvements. The machine learning algorithms used also achieved very high accuracy using Decision Tree and Gaussian Naive Bayes. This anti-stunting education model can be applied to the same case in a wider scope by paying attention to several aspects as an evaluation.

## 1 Introduction

Helminth infections contribute to 10–20 % prevalence among preschool children worldwide. Damage to the integrity of the intestinal barrier and decreased gastric acid secretion make the gastrointestinal tract more susceptible to pathogens, including parasites such as helminths [1]. These infections are a major factor that increases the risk of stunting. Globally in 2020, it is estimated that 149 million children under the age of 5 will be stunted, with significant geographical variations across different regions of the world [2]. The prevalence of stunting in Southeast Asia in children under five years old reached 24.7 % in 2018. In Indonesia, the figure was 30.8 %, although it decreased to 27.7 % in 2019, the issue remains a serious concern [3]. Improved health is considered an important aspect of well-being, and child health is known to have a long-term impact on productivity in adulthood [4].

Stunting results from chronic and recurrent undernutrition, characterized by a child having a shorter height than appropriate for his or her age, which results in impaired physical and cognitive growth [5], as well as a lack of stimulation during a child's formative years, especially in the first 1000 days of life (from pregnancy to 2 years of age) [6]. Stunting affects not only physical growth but also cognitive development and long-term health of the child.

According to the WHO, the definition of malnutrition is based on several measurements, including weight, length or height, and, more recently, upper arm circumference. These measurements are used separately or in combination, as in weight for height. The most common measurement is weight, which is usually adjusted for age through table look-ups, chart mapping, or converted to a standard deviation (z) score. A z-score for weight-for-age (WAZ) < −2 is defined as moderate malnutrition, while WAZ < -3 is considered severe malnutrition. A z-score for length-for-age (LAZ) < −2 was defined as stunting [7]. The WHO growth charts project has released age-standardized norms, as well as age- and sex-specific norms, for rates of weight gain over 4 and 8-week intervals [8]. They state that these growth rate measurements are very useful in predicting future stunting [9].

Nutrition interventions for children generally take place during the first two years of life, a period in which the prevalence of failure to thrive, micronutrient deficiencies and infectious diseases is particularly high in developing countries. After the age of two, the challenge of reversing the impact of undernutrition on stunting increases significantly, with the possibility of some functional deficits becoming permanent [10]. Nutrition interventions that have been shown to be effective include nutrition supplementation for pregnant and lactating women (PBW), counseling for mothers on infant and young child feeding (IYCF), and nutrition supplementation for children aged 6–23 months. A large body of evidence shows that nutrition supplementation, both with and without nutrition education, is effective in preventing and addressing stunting in developing countries [[11], [12], [13], [14], [15], [16], [17]].

Based on several types of effective nutrition interventions, this study approaches the provision of supplementation to breastfeeding mothers, infant and young child counseling and feeding, and nutrition supplementation for children under two years of age [10]. One intervention model that is not widely practiced in handling stunting is anti-stunting education with an approach to Islamic values as a local community belief. Islam through the Qur'an introduces itself as a guide for humans regardless of differences. Then, the application of the values of the Qur'an and Hadith as the main source of law in Islam becomes a key theological review to help deal with this case by covering all aspects of life.

The integration of Islamic values in this study is grounded in both contextual and scientific relevance. In Indonesia, where more than 85 % of the population is Muslim, religion plays a central role in shaping family health behaviors, dietary practices, and parenting norms. The Qur'an and Hadith emphasize cleanliness (thaharah), moderation in eating (wasatiyyah), and the responsibility of parents in nurturing and protecting their children's health. These principles directly align with key determinants of stunting such as hygiene, nutrition, and caregiving practices. From a scientific perspective, behavioral interventions are more effective when they align with the community's belief system, as culturally and spiritually congruent education increases acceptance, adherence, and sustainability of health programs. Therefore, integrating Islamic values in anti-stunting education provides both a culturally grounded and evidence-based pathway to promote healthier behaviors. This makes the intervention not only driven by religious motivation and the Qur'an [18], but also scientifically justified in the context of the socio-cultural reality of Indonesian communities.

As attention to the issue of stunting increases worldwide, researchers and health experts continue to look for ways to detect and prevent this condition early. One method that is increasingly being used is machine learning as AI-based modeling in predicting diseases including stunting [19], a technology capable of analyzing big data and identifying patterns that may not be visible to traditional methods. With these capabilities, machine learning provides new opportunities in predicting stunting, which is one of the major challenges in global child health. Machine Learning enables systems to learn from data without the need for explicit programming. This empowers machines to identify patterns within data and make decisions or predictions independently of direct human intervention. By leveraging algorithms and statistical models, Machine Learning improves its performance as it gains more experience and processes larger datasets. Its application in the medical field holds immense potential for disease prediction [20].

While interventions in children under two years of age are in focus, long-term studies evaluating the sustained effects of these interventions up to a certain age are limited. Research indicates that prolonged childhood malnutrition leads to long-term economic, social, and human impacts, such as stunted physical growth, reduced educational attainment, cognitive deficits, and diminished work productivity [19]. In addition, many nutrition interventions are generic, but there is a lack of research highlighting the importance of tailoring interventions to local contexts. This customization can consider cultural, social and economic factors, particularly with the integration of religious values that are relevant for Indonesia's diverse population.

From a theoretical perspective, stunting prevention interventions are based on behavioral and ecological models that view child growth as the result of interactions between individual, family, and environmental factors. Stunting occurs due to inadequate food intake, infections, poor hygiene practices, and limited parental knowledge, all of which are influenced by broader socio-economic and cultural conditions. Effective stunting prevention therefore requires interventions that integrate nutrition education, hygiene behavior, and motivation within the family environment. Educational efforts that strengthen mothers' awareness, parenting attitudes, and daily hygiene practices have been shown to improve children's growth when supported by consistent community engagement. The incorporation of religious or value-based elements, such as Islamic teachings promoting cleanliness, moderation, and balanced nutrition, further enhances behavioral adherence and sustainability.

In addition, the theoretical foundation of this study draws from health informatics, where machine learning is increasingly used as a decision-support system for identifying health risks and predicting disease patterns. Machine learning models such as Decision Tree and Naïve Bayes can recognize complex patterns in multidimensional health data and transforming them into predictive insights. Their application in stunting analysis allows for more precise identification of risk factors and supports data-driven decision-making for early intervention. Combining these analytical models with educational and behavioral interventions creates an integrated approach that connects predictive technology with practical community health strategies.

Although several studies have applied machine learning techniques to predict stunting or related health outcomes in children [21,22], most of these models focus solely on biomedical or demographic variables without incorporating context-specific sociocultural factors. Prior models often use limited datasets and rarely integrate intervention-based outcomes, such as the impact of religious or value-based education in mitigating stunting risks. Furthermore, existing research generally centers on classification tasks (e.g., predicting stunted vs. non-stunted) [[22], [23], [24], [25]] but lacks exploration into the longitudinal effects of early interventions or localized models tailored to specific populations such as Muslim-majority regions. This leaves a significant research gap in developing culturally adaptive and intervention-aware ML models for stunting prediction.

To address this gap, the current study introduces a dual innovation that distinguishes it from conventional stunting interventions. First, it embeds Islamic value–based education not merely as a religious perspective but as a structured behavioral-change framework, operationalized through measurable intervention dimensions motivation, hygiene, nutrition fulfillment, mental health, and infant health that collectively influence family and community health behaviors. Second, it employs machine learning not only as a classification tool but also as an analytical framework to evaluate how these behavioral and socio-religious factors interact with biomedical predictors to influence stunting outcomes.

This methodological integration bridges the domains of public health intervention and data-driven prediction, creating a culturally grounded yet technology-enabled model of stunting prevention. Unlike prior studies that rely primarily on anthropometric and demographic data, this research advances a culturally adaptive and intervention-aware ML model capable of capturing both biological and behavioral determinants of stunting. By aligning Islamic educational values with data analytics, the study contributes a novel, context-sensitive approach that enhances predictive accuracy while increasing community acceptance and ethical relevance in Muslim-majority contexts such as Indonesia.

This study aims to address the issue of stunting, a pressing public health concern that has become a top priority in the national health agenda [26] by leveraging a multidisciplinary and technology-driven approach. The widespread distribution of under-five children across various regions necessitates timely and accurate insights for policymakers to effectively predict and intervene in stunting cases. While most existing interventions primarily focus on supplementary feeding, alternative models - particularly those rooted in local socio-cultural values such as Islamic principles - remain underexplored. Therefore, this research integrates machine learning techniques to identify trends and classify stunting-related data, providing a robust foundation for more contextual and targeted interventions. By combining public health, data science, and culturally adapted strategies, the study seeks to offer scalable solutions that can be applied to similar settings facing stunting-related challenges.

While several previous studies have utilized machine learning to predict stunting [22,[27], [28], [29]], they often rely solely on clinical or demographic [[30], [31], [32]] variables and lack integration with intervention outcomes or socio-religious dimensions. This study introduces a novel framework by combining early nutritional intervention data and anti-stunting education based on Islamic values with machine learning algorithms. To the best of our knowledge, this integration of culturally grounded behavioral components into predictive modeling remains underexplored in current literature. The proposed model not only classifies stunting risk among children under five but also captures the contextual relevance of religious values in shaping family health behaviors. This approach contributes to the development of locally relevant, adaptive, and ethically grounded AI solutions in public health - particularly in Muslim-majority regions like Indonesia.

Despite numerous efforts to reduce stunting through nutritional and behavioral interventions, the persistence of high prevalence rates in Indonesia indicates that existing models remain limited in addressing cultural and contextual determinants. Most stunting interventions focus on biomedical and demographic aspects without considering behavioral, religious, and sociocultural factors that influence child nutrition and caregiving practices. Furthermore, predictive models based on machine learning have rarely integrated these multidimensional variables, resulting in limited contextual relevance and policy applicability. Therefore, the core problem addressed in this study is how to develop an intervention model that integrates Islamic value-based education with machine learning analysis to more accurately predict and reduce stunting among children under five in Indonesia.

The motivation for applying machine learning in this context stems from the limitations of conventional statistical methods in handling the complex, multifactorial nature of stunting. Traditional models often struggle to capture the nonlinear interactions between nutritional status, socio-demographic conditions, cultural practices, and behavioral change. Machine learning, in contrast, allows for automated pattern recognition and predictive modeling based on large, multidimensional datasets. This is particularly valuable in early identification of at-risk children and optimizing intervention strategies. Given the urgency of reducing stunting rates and the constraints on healthcare resources in Indonesia, the use of ML adds significant value by enhancing prediction accuracy, supporting evidence-based planning, and enabling more efficient allocation of interventions at scale.

Further research exploring this area will help develop more comprehensive, adaptive and evidence-based interventions to reduce stunting more effectively. The primary outcome of this study is to estimate the impact of supplementary feeding and anti-stunting education interventions on stunted children under 5 years of age in the defined working areas. Secondary study outcomes include modeling stunting and non-stunting data using machine learning on all children under the age of five in the measured age groups.

The remainder of this paper is organized as follows. Section 2 explains the research methodology, including study design, intervention strategies, and machine learning modeling. Section 3 presents the results of both statistical analyses and predictive modeling. Section 4 discusses the findings in relation to prior studies, highlighting the implications of integrating Islamic values and machine learning for stunting prevention. Finally, Section 5 provides conclusions, identifies the study's limitations, and suggests directions for future research.

The types of data collected in this study were directly aligned with the research objectives. Anthropometric measurements (height, weight, head circumference, and mid-upper arm circumference/MUAC) were used to identify and classify stunting cases as the foundation for developing predictive machine learning models. Meanwhile, questionnaire and interview data provided behavioral, socio-economic, and value-based insights related to nutrition, hygiene, and parenting practices. These qualitative and quantitative datasets were integrated to evaluate the effectiveness of Islamic value–based educational interventions and to strengthen the predictive features used in data mining models. Therefore, each data component biophysical, behavioral, and contextual was collected intentionally to support the studys goal of creating an evidence-based and value-oriented model for stunting prevention.

## 2 Method

To improve the quality of reporting in observational studies, we developed guidelines in the form of a checklist covering important aspects that need to be reported, known as the STROBE Statement (Strengthening the Reporting of Observational Studies in Epidemiology) [33]. The quantitative component assessed anthropometric changes, statistical differences between pre- and post-intervention outcomes, and the performance of machine learning algorithms. The qualitative component explored contextual, behavioral, and socio-cultural factors influencing intervention adoption through semi-structured interviews with key community stakeholders.

The mixed-methods approach was adopted for two reasons:

(1) To offer numerical and statistical evidence of intervention effectiveness, and.
(2) To capture the lived experiences, behavioral constraints, and community perceptions required to interpret quantitative findings and guide model refinement.

### 2.1 Quantitative

#### 2.1.1 Study design

The research aimed to answer three main questions: (1) What significant improvements occur in stunted toddlers following the intervention? (2) Is an intervention approach based on Islamic values effective in addressing stunting? (3) How accurate is the machine learning model developed to identify stunting cases? Quantitative data were collected from February to August 2024 using health records and anthropometric data from local health institutions. Meanwhile, The study also included a machine learning component (Naïve Bayes and C4.5 algorithms) to classify stunting risk, followed by interventions such as supplementary feeding, training, and socialization, which incorporated Islamic teachings derived from the Qur'an and Hadith (Table 5), and measured quantitatively through binary variables to assess its relationship with stunting status. Each intervention aspect was coded as a binary variable (Yes/No), indicating whether a participant attended or received a specific intervention, as reflected in Table 6: Variables in STROBE. These variables were used in two ways:

- Statistical analysis (pre-test and post-test): To evaluate the effectiveness of Islamic value-based interventions, we measured outcomes such as changes in stunting knowledge, attitudes, and actual stunting prevalence before and after the intervention (see Table 9, Table 10, Table 11).
- Model analysis (indirect evaluation): While the initial ML models did not include the intervention variables as input features, we conducted post-hoc analysis to examine the relationship between ML-predicted stunting status and intervention participation. Table 12, for example, shows statistically significant associations between participation in hygiene, nutrition fulfillment, and mental health sessions and improved stunting outcomes (*p* < 0.05).

This design enabled a hybrid approach: using ML to classify risk and guide targeted intervention and using traditional statistical analysis to measure intervention impact. The decision to exclude intervention variables from the initial predictive model was made to avoid data leakage and ensure the ML algorithms relied only on pre-intervention predictors. However, future models are planned to incorporate these behavioral and religious context features to assess whether culturally grounded variables improve predictive power.

**Fig. 1:**
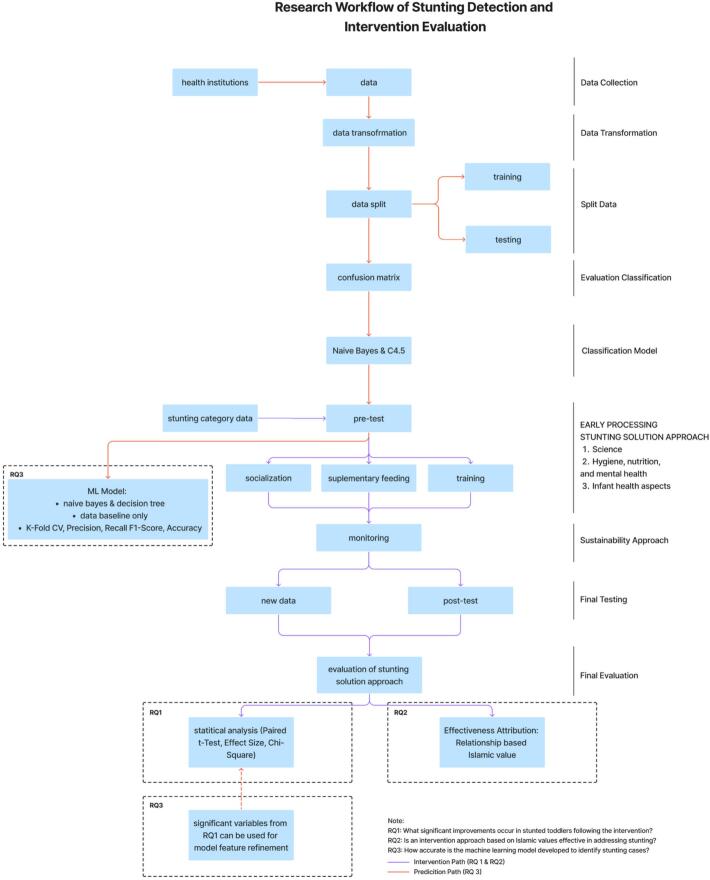
Reasearch flow diagram.

Fig. 1 shows methodological framework comprises two interconnected analytical paths.

(1) Intervention Path (addressing RQ1 and RQ2)

Baseline measurements (pre-test) obtained from health institutions were followed by a structured intervention grounded in Islamic values, encompassing motivation, hygiene, nutrition, mental health, and infant health components. Post-intervention data (pos*t*-test) were collected after a monitoring phase. Statistical analyses—including paired t-test, chi-square test, effect size calculation, and confidence intervals—were performed to evaluate significant changes (RQ1). Furthermore, an effectiveness attribution analysis was conducted to examine the relationship between observed improvements and the Islamic values-based components of the intervention (RQ2).

(2) Prediction Path (addressing RQ3)

Baseline data were also utilized to develop a predictive model for stunting classification. The dataset underwent transformation and was divided into training and testing subsets for machine learning algorithms (Naïve Bayes and C4.5 decision tree). Model performance was assessed using k-fold cross-validation, precision, recall, F1-score, and accuracy.

A feedback mechanism was established between the two paths; whereby significant variables identified from the intervention analysis (RQ1) were incorporated into the feature refinement process of the predictive model (RQ3). This integration ensured methodological coherence between empirical impact evaluation and predictive analytics.

Table 1 shows the quantitative operational definitions for data collection. These operational definitions explain the quantitative variables used in this study. These variables are definitions used in health sciences, religion, and informatics as this study involves multidisciplinary sciences.

**Table 1:** Operational definition quantitative.

Variable	Description
Target children	children 6–59 months of age
Wasting	toddlers with a nutritional status based on the indicators BB/PB or BB/TB at −3 SD to < −2 SD
Stunting	condition of impaired growth in children caused by chronic malnutrition over a long period, especially during the first 1000 days of life
Malnutrition	a general term for a condition of unbalanced nutrition both under and over nutrition.
Machine Learning	learn from data and make predictions or decisions without being explicitly programmed. Machine learning systems recognize patterns in data and use those patterns to classify, predict, or take action.
Underweight	toddlers with nutritional status based on BB/U or below -2SD
Severe pregnant mothers	pregnant women who have a pre-pregnancy Body Mass Index or in the 1st trimester (< 12 weeks) of <18.5 kg/m2 and or LiLA (MUAC) < 23.5 cm

Table 2 shows the Standard Local Supplementary Food for Toddlers that was used as one of the interventions. The food is rich in animal summer with attention to balanced nutrition, using fresh food ingredients (without artificial preservatives), limiting the consumption of sugar, salt and fat.

**Table 2:** Local supplementary food standards for toddlers.

Nutrient substance	Age			
	6–11	9–11	12–23	24–59
Calories (kcal)	175–200	175–200	225–275	300–450
Protein (gr)	3,5–8	3,5–8	4,5–11	6–18
Fat (gr)	4,4–13	4,4–13	5,6-17,9	7,5-29,3

#### 2.1.2 Setting

1) Location and time of the study

The study was conducted in six selected villages in East Lombok Regency, Indonesia - namely Denggen, East Denggen, Majidi, Sekarteja, Pancor, and Rakam. These villages were chosen due to their high prevalence of stunting and varied socio-cultural characteristics. Data collection took place from February to August 2024, involving collaboration with local health institutions, village officials, and community health workers. The interventions were carried out in community health posts (Posyandu) and village halls, making use of accessible community spaces to reach mothers and caregivers of stunted toddlers. The socio-religious context of the region also allowed the integration of Islamic values into the health education component, aligning with the community's beliefs and practices.

This research was carried out in areas that have a high rate of stunting in children under the age of five. East Lombok is ranked second out of 10 districts/cities in West Nusa Tenggara as shown in Table 4. The location was selected based on stunting prevalence data obtained from local and national health reports and considering cultural and socio-economic diversity. The selected location must have access to health facilities and community organizations that are committed to supporting nutrition interventions and anti-stunting education based on Islamic values. This aims to ensure the effectiveness and acceptance of interventions in different local contexts.

Table 3 presents a comparative overview of stunting prevalence at three administrative levels: national, provincial, and city. At the national level, among approximately 15.9 million children under five, 967,069 were classified as stunted, resulting in a prevalence rate of 6.1 %. At the provincial level, the prevalence is notably higher, with 55,810 out of 441,000 children (12.7 %) experiencing stunting. At the city level - the specific focus area of this study - the stunting rate reaches 15.9 %, with 19,642 out of 123,668 children affected. This table highlights a critical finding: the closer the analysis moves toward the local context, the higher the prevalence appears, underscoring the urgency of tailored, localized interventions to effectively combat stunting.

**Table 3:** Comparisons of stunting in national, province, and city.

	Baseline	Stunting	Prevalence
National	15,910,944	967,069	6.1
Province	441,000	55,810	12.7
City	123,668	19,642	15.9

Table 4 shows data obtained in 2023 from the West Nusa Tenggara Provincial Government through the Health Office. East Lombok ranks first based on the number of stunting cases among other districts/cities in West Nusa Tenggara Province with 19,567 cases.

**Table 4:** Prevalence stunting at West Nusa Tenggara, 2023.

No	City	Number of children under five measured	Number of stunted toddlers	Percentage of stunting
1	West Lombok	60,346	7468	12.38
2	Central Lombok	93,270	12,446	13.34
3	**East Lombok**	**120,932**	**19,567**	**16.18**
4	Sumbawa	34,028	2883	8.47
5	Dompu	20,901	2276	10.89
6	Bima	40,223	4737	11.78
7	West Sumbawa	11,433	874	7.6
8	North Lombok	21,513	3878	18.03
9	Mataram City	25,282	3732	14.76
10	Bima City	11,627	1441	12.39
Total		439,555	59,302	13.49

**Fig. 2:**
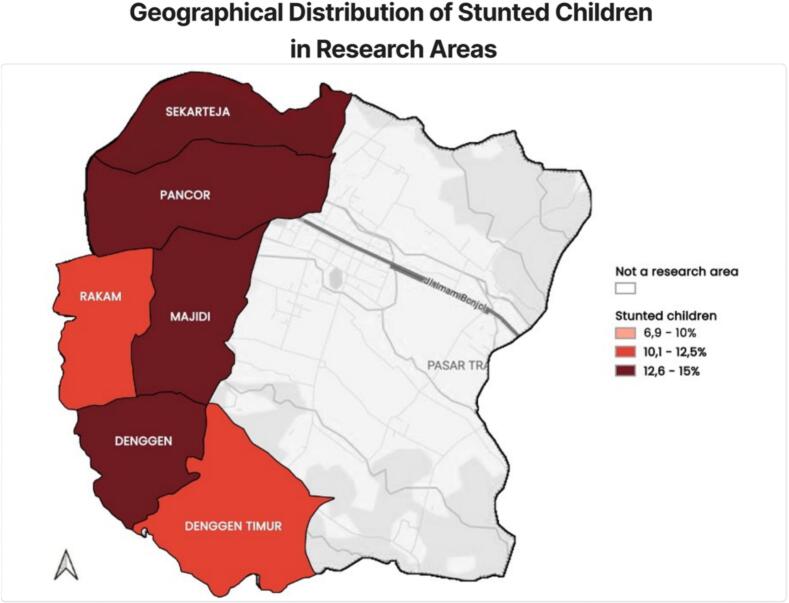
A map of six villages in East Lombok district.

Based on Table 3, Table 4, which show the prevalence of stunting nationally and provincially, this study finally took the locus of research in the East Lombok region, which is the region with the highest stunting rate in West Nusa Tenggara Province, the eastern province of Indonesia. More specifically, we took the Denggen area, as shown in Fig. 2.

Fig. 2 is data from February 2024 showing six villages in East Lombok that have high stunting status, namely Denggen Village, Denggen Timur (East Denggen), Majidi Village, Pancor Village, Rakam Village, and Sekarteja Village.

2) Implementation of the intervention

The intervention was carried out by the research team in collaboration with posyandu cadres, Puskesmas officers, and local religious leaders. Each implementer had a specific role:

a) Cadres and health workers guided PMT activities, nutrition education, and health checks.
b) Religious leaders provide education on Islamic values-based parenting.
c) The research team coordinated all activities and monitored the implementation of the intervention.

The intervention was carried out on toddlers at the maximum age of 24 months because the age group of toddlers who exceed this limit is one of the age group categories that is difficult to intervene [34]. Global and regional experts are working to reduce stunting in the “first 1000 days of life” through proven nutrition interventions. These include nutritional supplementation for pregnant and lactating mothers, education on infant and young child feeding practices (IYCF), and nutritional supplementation for children 6–23 months of age [10]. Therefore, we tried to conduct an experiment in the form of Supplementary Feeding, as well as education such as knowledge approaches, hygiene, fulfillment of nutrition and mental health, and aspects of infant health.

**Fig. 3:**
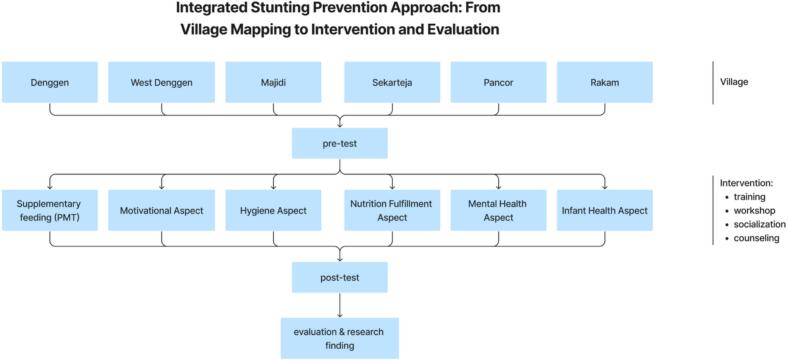
Intervention flowchart.

Fig. 3 shows the flowchart of the intervention provided to the six villages from pre-test, intervention to post-test. Furthermore, a detailed description of each intervention conducted is described in Table 5.

**Table 5:** Intervention description.

Intervention aspects	Method	Description
Supplementary feeding (PMT) [35,36]	Socialization & Counseling	a. Distribution of fortified porridge (200 g) 4×/week b. Nutrition counseling session per group of mothers c. Nutritional counseling: emphasizing QS Al-Baqarah [2]:233 on children's nutritional rights, and Hadith “A strong believer is better…” (HR Muslim) to motivate balanced intake
Motivational Aspect [37,38]	Workshop & Socialization	d. 2× parental resilience & goal-setting workshop e. Weekly motivational socialization f. Relates QS At-Taubah [9]:105 (“…I created…”): instilling the belief of effort + prayer, as well as the Hadith about tawakkul (sincerity) as the key to family success.
Hygiene Aspect [39,40]	Training & Socialization	g. Hand washing practice h. Socialization of latrine sanitation (1 session per village) i. Linking the Hadith “Thahārah (purification) is part of faith” (HR Muslim) and QS Al-Ma'idah [5]:6 (ablution) to reinforce clean living behavior
Nutrition Fulfillment Aspect [41,42]	Demo & Training	j. Demonstration of cooking a balanced menu k. Distribution of balanced nutrition leaflets l. Nutrition leaflet: includes QS Al-Mā'īdah [5]:88 (“…and eat good food”) and the Hadith on the prohibition of excess (‘Isrāf’) so that the diet is proportionate.
Mental Health Aspect [43,44]	Extension & Counseling	m. 3× mental health counseling session n. Mother's group psychosocial counseling o. Integrating QS Al-Baqarah [2]:155 (“…indeed we will test you…”): fostering patience, and the Hadith “indeed with difficulty there is ease” (QS Al-Inshirah).
Infant Health Aspect [45,46]	Training & Socialization	p. Early Breastfeeding Initiation Training (IMD) q. Socialization of basic immunization program r. Immunization socialization: referring to QS Al-Anbiyāʾ [20]:79 on child protection, and Hadith “the best among you are…” (HR Al-Bukhari) regarding the role of fathers and mothers.

Table 5 summarizes a holistic intervention strategy to address stunting through six integrated aspects: supplementary feeding, motivation, hygiene, nutrition fulfillment, mental health, and infant health. Each intervention combines practical methods such as counseling, training, and demonstrations with Islamic values from the Qur'an and Hadith to strengthen community acceptance and internal motivation. For example, nutrition counseling is paired with QS Al-Baqarah [2]:233, while hygiene training draws from QS Al-Ma'idah [5]:6. This value-based approach aims to enhance both the scientific and spiritual dimensions of behavioral change in preventing stunting.

3) Duration and frequency of intervention
  - Activities are conducted once a week (±12 meetings in total).
  - Health checks are conducted once per month.
  - Each education and training session lasts about 1–1.5 h per meeting.
4) Media and method of implementation

The media used include leaflets, educational modules, posters, healthy food props, and educational videos. The methods used were interactive lectures, demonstrations, group discussions, parenting simulations, and hands-on practice of supplementary feeding. All materials were adapted to the local cultural context and language understood by the participating mothers.

#### 2.1.3 Participants

1) Study sample

The quantitative sample consisted of children under the age of two who were identified as stunted based on WHO standards, along with their mothers or guardians who served as respondents in the nutrition and education interventions. Inclusion criteria were: (1) toddlers diagnosed with stunting, and (2) families willing to participate in the intervention program. Exclusion criteria included toddlers with other health conditions affecting growth and development or families unable to participate consistently during the study period. Sampling was conducted purposively, considering representation and alignment with intervention goals.

2) Participants recruitment

Recruitment was carried out in collaboration with local health service centers—including the district health office, health centers (Puskesmas), and Posyandu—together with village governments. These institutions disseminated information about the study and its objectives to eligible families. Mothers who expressed interest received detailed explanations regarding study procedures, duration, and potential benefits for their children. Confidentiality was ensured, and written informed consent was obtained from all participants before data collection.

3) Data collection

Quantitative data were collected by trained nutritionists and local government health workers to ensure accuracy and compliance with research ethics. Ethical approval was granted by the Research Ethics Committee of Ahmad Dahlan University (Approval No. 012406159). A portion of anonymized quantitative data has been published in the Mendeley Data repository (DOI: 10.17632/z4mrz37dm3.2). Anthropometric measurements—including arm circumference, head circumference, height/length, and weight—were collected from children aged 0–60 months before and after the intervention to enable pre–post comparison. Mothers or guardians also completed a structured questionnaire using a 5-category scale scored from 1 to 3 (1 = yes, we have this; 2 = we are working on it; 3 = no, we do not have this).

#### 2.1.4 Variables

As for the quantitative variables, they are as follows:

1) Explanation of quantitative variables used (age, weight, height, head circumference, etc.)
2) Whether categorization of numerical variables was done (age categorized as 0–11 month, 12–23 month, etc.)
3) Adjustments for analysis (normalization for machine learning models)

Table 6 outlines the variables used in the study based on the STROBE framework, categorized into outcomes, interventions, and predictors. The main outcome is stunting status, defined by WHO standards (z-score < −2). Interventions include several binary variables such as supplementary feeding, nutrition education, Islamic parenting education, hygiene training, maternal motivation, and health check-ups. Predictor variables include child-specific metrics (age, gender, weight, height, arm and head circumference) and maternal or household factors like education level, socioeconomic status, and Islamic literacy, enabling comprehensive analysis of factors influencing stunting.

**Table 6:** Variable in STROBE.

Category	Variable	Definition (simplified)	Scale
Outcome	Stunting Status	WHO: z-score < −2 = Stunted	Stunted / Not Stunted
Intervention	Supplementary Feeding	Given additional nutritious food	Yes/No
	Nutrition Education	Mother joined nutrition sessions	Yes/No
	Islamic Education	Parenting with Qur'an & Hadith values	Yes/No
	Hygiene & Sanitation	Hygiene training provided	Yes/No
	Maternal Motivation	Training to boost caregiving motivation	Yes/No
	Health Check-Ups	Routine visits to health center	Yes/No
Predictor/Covariate	Toddler's Age	Age in months	Numeric
	Gender	Child's gender	Male / Female
	Body Weight	Measured in kg	Numeric
	Height	Measured in cm	Numeric
	Arm Circumference	Mid-upper arm in cm	Numeric
	Head Circumference	Head size in cm	Numeric
	Mother's Education	Education level achieved	No School – Higher Ed
	Socioeconomic Status	Income & asset level	Low / Middle / High
	Islamic Literacy	Score from Islamic knowledge questionnaire	Good / Moderate / Poor

Intervention variables such as PMT, nutrition education, Islamic education, hygiene training, maternal motivation, and health checks were coded binarily for statistical analysis, but were not used as input features in the initial machine learning model. The ML model only uses baseline numerical variables such as age (months), weight (kg), height (cm), upper arm circumference (cm), and head circumference (cm), as well as coded categorical variables such as gender and maternal education. This approach was chosen to avoid data leakage and ensure the model reflects predictions based on initial conditions prior to intervention.

#### 2.1.5 Data source/measurement

The data used in this study are primary data collected directly through:

1) Anthropometric measurements (weight, height, upper arm circumference, etc.) by trained health workers.
2) Structured questionnaires to explore information on maternal background, socioeconomic status, and Islamic literacy level. A pre-test is conducted before the intervention begins to measure initial or baseline conditions. At this stage, data on the respondents' initial state or understanding (e.g., knowledge, attitude, or behavior related to stunting) is collected. The aim is to get an initial picture that will be the basis for comparison after the intervention. While the post-test is conducted after the intervention is completed, to see the changes that occur because of the intervention. The data collected in the post-test is compared with the pre-test results to evaluate the extent to which the intervention has had a positive effect or the desired change.
3) To evaluate the effectiveness of the intervention, this study used a pre-test and post-test design. The pre-test was conducted in February 2024 before the intervention was carried out, involving all toddlers experiencing stunting and their mothers or caregivers at health posts and village halls. Trained health workers collected anthropometric data (weight, height/length, MUAC, head circumference), and mothers/caregivers completed structured questionnaires on knowledge, attitudes, and practices related to stunting prevention, hygiene, nutrition, mental health, and infant care, with attendance and consent recorded. Post-intervention tests, conducted in August 2024 after a 12-week intervention, repeated these procedures and documented participation in supplementary feeding, motivational sessions, hygiene training, nutrition workshops, mental health counseling, and infant health programs. In this paired design, the pre-intervention test provided baseline data for statistical analysis and machine learning modeling, while the post-intervention test captured changes attributable to the intervention, enabling a clear, measurable, and reproducible evaluation of the intervention's effectiveness.
4) Documentation of intervention activities such as attendance lists, counseling notes, and field observations.

All data were coded and inputted into Microsoft Excel, then cleaned and processed using SPSS for statistical analysis and Python (Scikit-learn) for machine learning analysis.

#### 2.1.6 Bias

1) Information Bias: Prevented by enumerator training, use of standardized measurement tools (e.g. calibrated anthropometric tools), and pretested questionnaire instruments.
2) SMOTE: A technique used to handle data imbalance between classes in machine learning.

#### 2.1.7 Study size

The study involved a total of 60 mother-infant pairs who met the inclusion criteria. This number was determined purposively based on the availability of the population in the intervention area and the capacity of the intervention for 3 months. The determination of this number also considered the effectiveness of the program and the minimum validity for predictive analysis with machine learning.

#### 2.1.8 Machine learning analysis

1) Data Processing
  a) Data Cleaning
  All anthropometric data (age, weight, height, upper arm circumference, head circumference) were checked for outliers and inconsistencies. Unrealistic extreme values were verified and excluded if invalid.
  b) Handling Missing Values
  Missing data were handled with different strategies according to the variable: listwise deletion for completely missing data, while median imputation was used for partially missing numeric data.
2) Imbalance Data Handling

Data imbalance often occurs in real-world applications such as medical diagnosis, pattern recognition, speech recognition, and fraud detection. Machine learning has the potential to lead to overfitting [47]. In classification datasets, the number of observations may be dominated by the majority class and only a few belong to the minority class [48]. There are various techniques to handle unbalanced data, such as oversampling, Synthetic Minority Over-sampling Technique (SMOTE), and under sampling. Under sampling involves randomly subtracting from the majority class (the class with the dominant amount of data) to balance the dataset. Oversampling techniques are important for retaining attributes and can also be used to fill in missing values. Meanwhile, SMOTE is an effective oversampling approach in dealing with unbalanced datasets [49]. This approach creates a new synthetic sample for the minority class by interpolating between the minority class data points [50]. SMOTE is also useful in preventing overfitting in machine learning algorithms [51].

3) Naïve Bayes

The Naive Bayes classifier is based on Bayes' theorem [52,53] and belongs to the supervised learning type [54]. This model indicates whether a class characteristic is considered independent [55] of other characteristics [56]. The main advantages of using the Naïve Bayes classifier are its ease of implementation and the speed at which it works [57,58]. This method is also growing in popularity thanks to its simplicity [59,60]. The simple probabilistic Naive Bayes method makes predictions based on class membership assumptions determined through Eq. (1) [61].

(1) $$P\left(A|B\right)=\frac{P\left(B|A\right).P\left(A\right)}{P\left(B\right)}$$

P(A|B) represents the conditional probability of event A occurring given that event B has occurred, while P(B|A) represents the conditional probability of event B occurring given that event A has occurred. P(A) denotes the probability of event A, and P(B) denotes the probability of event B.

4) Decision Tree C4.5

The C4.5 algorithm is an improvement of the ID3 algorithm [62], which can handle continuous attributes and use gain information as the basis for attribute selection, thus effectively overcoming the weakness of bias in attribute selection found in the ID3 algorithm [63]. The ID3 algorithm operates under the assumption that its hypothesis space includes every possible decision tree [64].

5) Evaluation Classification

Various performance indicators are applied to evaluate the effectiveness of each machine learning model. These metrics serve as objective benchmarks in assessing model performance [65]. Accuracy, recall, F-Measure, and precision are some of the important measures used [66]. Method evaluation in classification models can be used using Eqs. (2), (3), (4), (5) [67,68].

(2) $$\text{Accuracy}=\frac{\left(\mathrm{TP}+\mathrm{TN}\right)}{\left(\mathrm{TP}+\mathrm{TN}+\mathrm{FP}+\mathrm{FN}\right)}$$

(3) $$\text{Precision}=\left(\frac{\mathrm{TP}}{\mathrm{TP}}\right)+\mathrm{FP}$$

(4) $$\text{Recall}=\left(\frac{\mathrm{TP}}{\mathrm{TP}}\right)+\mathrm{FN}$$

(5) $$F1=2\;x\;\mathrm{precision}\;x\frac{\mathrm{recall}}{\mathrm{precision}}+\mathrm{recall}$$

where TP refers to a true positive result, FP to a false positive result, and FN to a false negative result.

Accuracy is a general measure that indicates the proportion of correct predictions among all predictions (Eq. (2)). However, in cases of class imbalance—such as in stunting classification where the minority class is crucial—accuracy alone can be misleading. For this reason, additional metrics are included.

Precision (Eq. (3)) quantifies the proportion of true positive results among all positive predictions, which is important to reduce false positives in health-related contexts. Recall (Eq. (4)), also known as sensitivity, measures the proportion of true positives correctly identified, and is particularly critical in this study to ensure that stunting cases are not overlooked. The F1-score (Eq. (5)) provides a harmonic mean of precision and recall, offering a balanced evaluation when the dataset is imbalanced.

To provide a more comprehensive assessment, the study not only reports the numerical values of these metrics but also interprets their implications for practical decision-making in stunting prevention. Specifically, higher recall values are prioritized, as missing a true stunting case (false negative) may lead to delayed intervention.

The analysis and presentation of results were structured in line with the STROBE framework and explicitly organized according to the three research questions (RQ1–RQ3) to ensure coherence and readability.

### 2.2 Qualitative

#### 2.2.1 Type of qualitative study

The qualitative component employed a descriptive qualitative design to explore contextual and behavioral factors influencing intervention adoption and community perceptions of stunting prevention. Qualitative data were gathered through interviews with participants specifically mothers of toddlers residing around the study sites.

Table 7 illustrates data collection with qualitative variables in the scope of health science and religion.

**Table 7:** Operational definition qualitative.

Variable	Description
Complete food	a complete nutritionally balanced meal consisting of staple foods, animal and vegetable side dishes, vegetables and fruit, accompanied by adequate water consumption
Breakfast	food that is not a main meal (breakfast, lunch, dinner) consumed between main meals that can help meet the adequacy of daily needs
Local food	foods consumed by local communities in accordance with the potential of local resources and wisdom and become an alternative source of carbohydrates, protein, fat, vitamins and minerals
Supplementary feeding (PMT)	Local food-based supplementary feeding (PMT) is the provision of local food-based supplementary feeding to improve the nutritional status of targets.
Motivational Aspect	Anti-stunting awareness, health awareness, understanding Qur'an
Hygiene Aspect	Clean self and environment
Nutrition Fulfillment Aspect	Eat and drink halal and good
Mental Health Aspect	Efforts to maintain mental health, then put your trust
Infant Health Aspect	Breastfeeding, birth spacing, Vaccinations, and paying attention to the growth child's development with love

#### 2.2.2 Qualitative participants

1) Qualitative Informants

The qualitative component involved key informants selected purposively from community health stakeholders. These included Posyandu cadres, Puskesmas officers, village health volunteers, and parents or guardians of toddlers who participated in the intervention.

2) Qualitative Data Collection

Semi-structured interviews were conducted face-to-face at Posyandu posts and health centers, lasting approximately 30–45 min. Interviews continued until information saturation was reached, ensuring no new themes emerged. Discussions explored challenges in nutritional fulfillment, hygiene practices, and behavioral change throughout the intervention. All interviews were audio-recorded with consent, transcribed verbatim, and analyzed using thematic analysis to identify recurring patterns and contextual insights that complemented the quantitative findings.

#### 2.2.3 Qualitative data analysis

A thematic approach was used. Transcripts were coded manually to identify recurring patterns and themes. Emerging themes were aligned with the quantitative findings to triangulate results, clarify behavioral influences, and contextualize statistical and predictive outputs.

#### 2.2.4 Ethical considerations

Ethical approval was granted by the Research Ethics Committee of Ahmad Dahlan University (Approval No. 012406159). All participants provided written informed consent prior to data collection. Confidentiality, voluntary participation, and the right to withdraw were ensured throughout the study. Data archived in the Mendeley Data Repository (DOI: 10.17632/z4mrz37dm3.2) adhered to ethical standards for human subject research.

## 3 Result

The data presented were obtained from measurements made by local health institutions to guardians/parents of children under five. The six villages included were the working areas of the health institutions. Ethical approval was taken from all guardians/parents of children under five involved. Questionnaires were used to collect information on complementary feeding practices, child anthropometric status, mental health, environment and socioeconomic status. The questionnaire was filled in before and after the intervention. To ensure coherence between the study design and the research outcomes, the results are presented in alignment with the three research questions stated in the methodology. RQ1 examines the changes in stunting status before and after the intervention, RQ2 evaluates the effectiveness of Islamic value-based interventions, and RQ3 assesses the predictive accuracy of machine learning models.

### 3.1 Participants and descriptive data

#### 3.1.1 Participants

A total of 3796 children under five from six villages in Selong Township, East Lombok Regency, were involved in the baseline phase of the study. The villages included Denggen, Denggen Barat, Majidi, Sekarteja, Pancor and Rakam. After the intervention process that lasted for several months, the number of children under five years old recorded at the endline stage was 3229 children. The main respondents in the data collection were mothers or primary caregivers of children under five, who had previously been screened through a questionnaire on basic literacy skills and commitment to stunting prevention. Most participants were housewives (76.9 %), aged ≥25 years (76.9 %), and had educational backgrounds at the junior and senior high school levels. Economically, most had an income of less than IDR 500,000 per month, reflecting the high economic vulnerability of the target group. In addition, many parents are migrant workers abroad and often leave their children with caregivers (family or neighbors).

Purposive sampling was applied to select 52 stunted children aged ≤24 months from a population of 3416 children aged 0–60 months identified during the August 2024 measurement. The choice of this age group was based on the critical “first 1,000 days of life” period, which represents the most sensitive developmental window for growth and cognitive formation. Intervening within this range allows more effective evaluation of stunting prevention through nutritional and behavioral interventions. Meanwhile, children older than 24 months were excluded because stunting cases in this age are generally more chronic and less responsive to short-term intervention.

Of the 3796 children initially recorded, 3416 had complete and valid anthropometric data that were used in the final analysis.

#### 3.1.2 Descriptive data

Data on pregnant women was collected from January 2023 to January 2024 in the region. Data collected during pregnancy plays a crucial role in determining the risk of stunting in children. Information on the mother's nutritional status, weight, and nutrient intake helps assess the mother's health condition, which greatly affects the growth of the fetus. In addition, a history of illness or complications during pregnancy, such as anemia or infection, and age can negatively impact fetal development and increase the risk of stunting.

The data studied is data on toddlers who have stunting status in February 2024 and August 2024. Measurements were taken twice to be able to intervene and make comparisons. Of the total 3416 children aged 0–60 months recorded during the measurement, 52 children aged ≤24 months were purposively selected for the intervention. This subgroup represents early stunting cases prioritized for nutritional and behavioral improvement efforts.

**Fig. 4:**
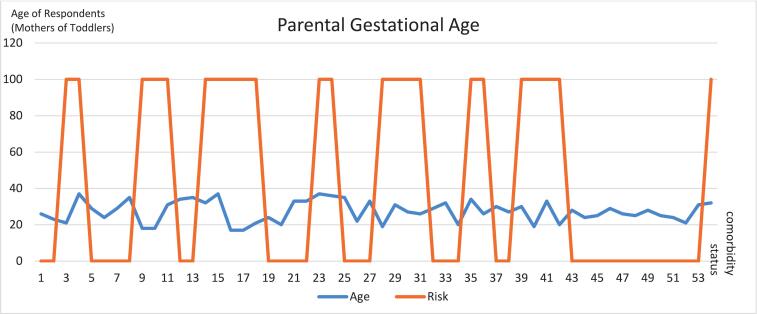
Data collection parental gestational age.

**Fig. 5:**
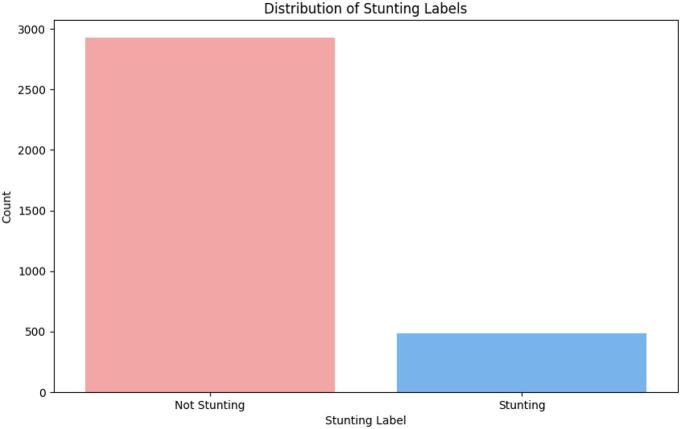
Distribution of stunting and not stunting.

Fig. 4 shows the age range of pregnant women for monitoring and allows early identification of developmental delays. The age range of pregnant women is 17 to 37 years old at the time of pregnancy and has a status of comorbidities, diseases such as mild anemia, heart disease, due to surgery, and gestational age categorized as young or old which is marked by giving a value of 100 on the graph. The data shows that 41.5 % of pregnant women have a high risk of stunting their children. Distribution of stunting and non-stunting in August 2024 among 3416 children shows in Fig. 5.

Fig. 5 shows the distribution of stunting and non-stunting in August 2024 among 3416 children.

Table 8 shows the results of respondent's ability screening aimed at understanding mothers' readiness and early awareness in preventing stunting. Most respondents (85.2 %) reported reading books, and all of them (100 %) read at least one book per week, which reflects good basic literacy. 77.8 % of respondents had implemented stunting prevention efforts, and most (55.6 %) reported implementing more than two important aspects, such as clean and healthy living behaviors (33.3 %), balanced nutrition (7.4 %), and attention to children's health (3.7 %). This finding supports intervention readiness, as most mothers already have a basic awareness of the stunting issue.

**Table 8:** Respondent ability screening.

Question	Answer			
Do you read books?	Yes	No		
	85.2 %	14.8 %		
Read a book in one week?	Yes	No		
	100 %	0 %		
Implementing Efforts in Stunting Prevention	Yes	No		
	77.8 %	22.2 %		
Things that are applied	Clean and healthy living behavior	Balanced nutrition	Child health	More than 2 points
	33.3 %	7.4 %	3.7 %	55.6 %

Table 9 presents the demographic characteristics of respondents in the study. Most respondents were ≥ 25 years old (76.9 %) and had a secondary education (69.2 % of junior and senior high school graduates). Economically, most had low incomes, with 80.8 % earning below IDR 500,000 per month. Most respondents were housewives (76.9 %) and all were married. Pregnancy history showed that 38.5 % had only been pregnant once, and most deliveries were normal (92.3 %). A total of 15.4 % of respondents had experienced anemia. This data shows that most mothers are of productive age but have economic limitations that can affect the nutritional and health conditions of their children.

**Table 9:** Demographic characteristics.

Characteristics	Group
Age	
<25 years	23,1 %
≥ 25 years	76,9 %

Education	
Not in school	3.8 %
Elementary	26.9 %
Junior high	34.6 %
High school	34.6 %

Income	
IDR <250,000	38.5 %
IDR 250,000 - 500,000	42.3 %
IDR500,000–1.000,000	15.4 %
IDR 1000,000 - 3,000,000	3.8 %

Occupation	
Self-employed	3.8 %
Housewife	76.9 %
Laborer	11.5 %
Other	7.7 %

Marriage Status	
Married	100 %
Widowed/Widower	0 %

Pregnancy History	
1 time	38.5 %
2 times	23.1 %
More than 2× pregnancy	23.1 %
2 points	15.4 %

Childbirth History	
Normal	92.3 %
Cesarean	7.7 %

History of Anemia	
Ever	15.4 %
Never	84.6 %

### 3.2 Outcome data

To address RQ1, Table 10, Table 11, Table 12 present the changes in stunting knowledge, attitudes, and prevalence before and after the intervention. (See Table 13.)

**Table 10:** Descriptive analysis.

Question	Pre-test		Post-test	
	Good	Not good	Good	Not good
Stunting knowledge	76.9 %	23.1 %	92.3 %	7.7 %
Stunting prevention attitude	61.5 %	38.5 %	73.1 %	26.9 %
Attitude behavior of stunting prevention	61.5 %	38.5 %	61.5 %	38.5 %

Note: The data in Table 10 represent the stunted group, which was the exclusive target of the intervention. Non-stunted children were not included in the treatment group; therefore, comparative results are not displayed.

**Table 11:** Comparison of pre- and post-intervention stunting status by age.

Villages	Age group									
	0–11		12–23		24–35		36–48		49- >60	
	Baseline	Endline	Baseline	Endline	Baseline	Endline	Baseline	Endline	Baseline	Endline
Denggen	2	0	12	8	22	15	14	15	20	11
	(13.3 %)	(0.0 %)	(12.9 %)	(16.3 %)	(14.0 %)	(30.6 %)	(11.3 %)	(30.6 %)	(19.2 %)	(22.4 %)
East Denggen	2	6	14	14	16	28	5	16	1	12
	(13.3 %)	(7.9 %)	(15.1 %)	(18.4 %)	(10.2 %)	(36.8 %)	(4.0 %)	(21.1 %)	(1.0 %)	(15.7 %)
Majidi	6	0	27	21	30	29	25	23	10	13
	(40.0 %)	(0.0 %)	(29.0 %)	(24.4 %)	(19.1 %)	(33.7 %)	(20.2 %)	(26.7 %)	(9.6 %)	(15.1 %)
Sekarteja	0	8	15	32	46	52	38	30	21	2
	(0.0 %)	(7.8 %)	(16.1 %)	(25.8 %)	(29.3 %)	(41.9 %)	(30.6 %)	(24.2 %)	(20.2 %)	(1.6 %)
Pancor	5	0	18	26	24	23	26	23	19	19
	(33.3 %)	(0.0 %)	(19.4 %)	(28.6 %)	(15.3 %)	(25.3 %)	(21.0 %)	(25.3 %)	(18.3 %)	(20.9 %)
Rakam	0	8	7	37	19	27	16	11	33	19
	(0.0 %)	(7.8 %)	(7.5 %)	(36.3 %)	(12.1 %)	(26.5 %)	(12.9 %)	(10.8 %)	(31.7 %)	(18.6 %)

**Table 12:** Comparison of pre- and post-intervention stunting status by village.

Villages	Baseline (N)	Stunting		Endline (N)	Stunting		*z-score*	*p-value*
		n	%		n	%		
Denggen	478	70	14.64	422	49	11.58	1.35	0.176
East Denggen	320	38	11.87	307	76	24.67	−4.16	0.0000318
Majidi	769	98	12.74	535	86	15.87	−1.565	0.118
Sekarteja	874	120	13.73	767	124	16.08	−1.384	0.1662
Pancor	729	92	12.62	664	91	13.62	−0.5986	0.5494
Rakam	626	75	11.98	515	102	19.73	−3.5976	0.000321
Total	3796	493	12.99	3229	528	16.92	−1.645	0.175

**Table 13:** Characteristics of interventions with parents or guardians of children.

Characteristic	Stunted	*p-value*
Motivational Aspect		
Consistent	180	0.214

Hygiene Aspect		
Hand washing	300	0.00099
Using clean water	450	0.00066
Using a toilet	430	0.00184

Nutrition Fulfillment Aspect		
Complete vaccine administration	400	0.000006
Breastfeeding	320	0.00726
Complementary feeding	370	0.02556

Mental Health Aspect		
Sleep disorders	150	0.00033
Anxiety disorders	100	0.11727

Infant Health Aspect		
Physical growth and development	220	0.22517
Intellectual growth and development	300	0.05450
Emotional growth and development	180	0.21377

Table 10 shows the results of descriptive analysis related to respondents' knowledge and attitudes before and after the intervention. Knowledge about stunting increased from 76.9 % (good) in the pre-test to 92.3 % in the post-test. Attitudes toward stunting prevention also increased from 61.5 % to 73.1 %. However, actual behavior in stunting prevention showed no change, remaining at 61.5 % which indicates a good attitude. This indicates that although the intervention was successful in improving understanding and attitudes, behavior change requires time and further approaches.

Table 11 presents a comparison of stunting status among under-fives before (baseline) and after (endline) the intervention by age group in the six villages. In general, there is a change in the proportion of stunting after the intervention, but the results vary by region and age group. For example, in Majidi for the 0–11 month age group there was a significant decrease from 40 % to 0 %, as well as in Denggen, East Denggen and Pancor. However, in contrast to Sekarteja and Rakam, where the stunting rate decreased between 36 and 48 and 49–60. This shows that although the intervention program has a positive impact on some age groups and regions, the results are not evenly distributed and are influenced by many factors such as the age of the child, environmental conditions, and the effectiveness of the intervention implementation in each region.

Table 12 shows the comparison of stunting status before (baseline) and after (endline) the intervention in six villages. In general, there was an increase in the percentage of stunting from 12.99 % to 16.92 % after the intervention, although not statistically significant overall (*p* = 0.175). Therefore, at the aggregate level, the intervention cannot be concluded to have a significant impact on reducing stunting prevalence. However, when analyzed at the village level, two villages—East Denggen and Rakam—showed a statistically significant increase in stunting (*p* < 0.001), while Denggen experienced a non-significant decrease. This indicates that the intervention outcomes varied substantially depending on local contextual factors rather than reflecting a uniform program effect across all areas.

The significant increase in stunting cases observed in East Denggen, Rakam, and Sekarteja indicates the presence of contextual and structural barriers that limited the intervention's effectiveness. These areas are geographically more distant from the community health center, resulting in less frequent supervision, limited access to clean water, and lower monitoring consistency. Moreover, household economic instability and parental migration—particularly fathers working outside the region—reduced daily childcare engagement, weakening the behavioral adoption of nutrition and hygiene practices. Cultural perceptions about child growth and feeding, such as delayed complementary feeding and reliance on traditional weaning foods, also persisted despite educational sessions. Additionally, these villages had lower cadre-to-household ratios and weaker coordination with local religious leaders, leading to lower community participation compared to Denggen. The combined influence of these logistical, socio-economic, and cultural constraints likely contributed to the increase in stunting prevalence, underscoring that short-term behavioral interventions are insufficient without addressing underlying systemic and environmental determinants.

To strengthen the analysis, effect size measurements and 95 % confidence intervals were added to key comparisons. For the paired *t*-tests assessing pre- and post-intervention knowledge and attitude (Table 10), we calculated Cohen's d. For instance, the increase in stunting knowledge (from 76.9 % to 92.3 %) yielded a Cohen's d of 0.68, indicating a moderate to large effect size. Similarly, for stunting prevention attitude (61.5 % to 73.1 %), the effect size was 0.51 (moderate).

For categorical data such as stunting status by village (Table 12), Cramer's V was used to assess the strength of association from the Chi-Square test. For example, in East Denggen (*p* < 0.001), the Cramer's V was 0.18, indicating a weak-to-moderate effect.

Confidence intervals (95 %) for the differences in stunting proportions before and after the intervention were also added. For instance, Denggen showed a change from 14.64 % to 11.58 %, with a 95 % CI of [−0.01, 0.07], confirming the change was not statistically significant. However, in East Denggen, the increase from 11.87 % to 24.67 % had a 95 % CI of [0.06, 0.18], confirming a significant worsening trend.

### 3.3 Main results

#### 3.3.1 Effects of intervention on stunting status

**Fig. 6:**
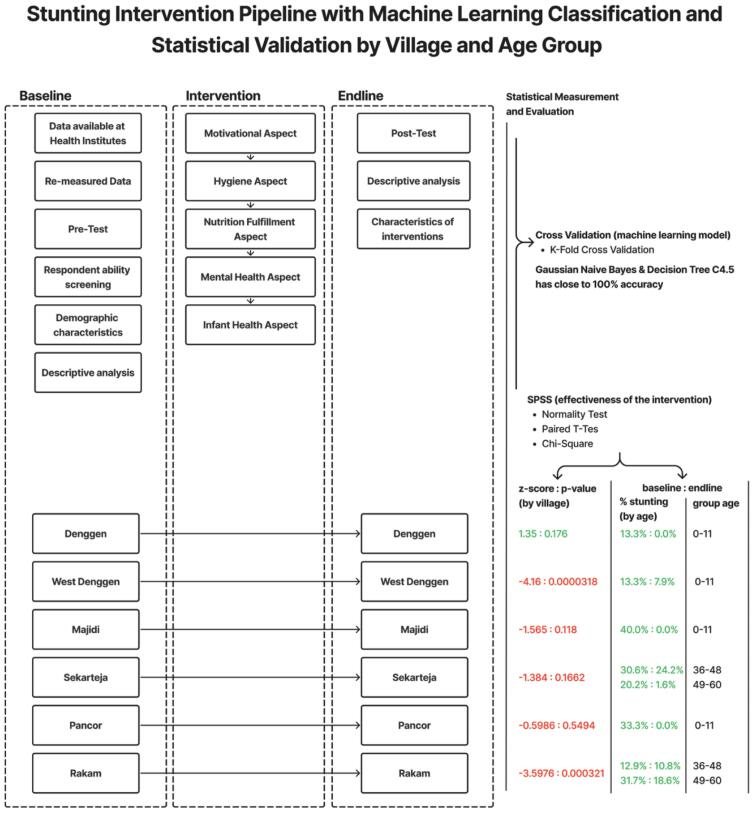
Main results in this research.

Based on Fig. 6, in general, the main results in this study show that Islamic values-based stunting interventions that cover aspects of motivation, hygiene, nutrition fulfillment, mental health, and infant health have a significant impact in reducing the prevalence of stunting in several intervention villages. The research process was conducted through three main stages: baseline, intervention, and endline, with evaluation using two approaches, namely machine learning (Naive Bayes and Decision Tree C4.5) which showed almost 100 % accuracy, and statistical analysis using SPSS through paired t-test and chi-square. The best results were found in Denggen by age and by village. Some areas other than Denggen did not experience a decrease by village, but 4 areas experienced a significant decrease by age with the under-five age category 0–11, and two others experienced a decrease in the under-five age groups 36–48 and 49–60. The process was measured using SPSS, while the machine learning model modeled the research workflow from baseline to endline. The machine learning model was evaluated using cross validation and the accuracy of the two machine learning methods used was close to 100 %.

In response to RQ2, intervention variables defined in the STROBE framework were analyzed for their association with stunting outcomes.

Calculations were performed using SPSS to analyze the collected data. Table 9 shows the demographic characteristics of mothers/parents of children under five. Table 8 presents the stunting status in the six regions by age before and after the intervention. Table 11 shows the stunting status by region before and after the intervention, while Table 12 highlights that aspects of hygiene, nutritional fulfillment, and some aspects of mental health demonstrated statistically significant differences, indicating distinctions between children who were and were not stunted. Conversely, aspects of infant health, such as physical and emotional growth, did not show significant differences. To ensure the robustness of the statistical evaluation, several tests were conducted. A normality test was first performed to determine whether the data followed a normal distribution, which is a prerequisite for using parametric tests. Subsequently, a paired *t*-test was employed to compare the means of variables before and after the intervention within the same group, aiming to assess the statistical significance of changes across key intervention aspects, including nutrition, hygiene, motivation, mental health, and infant care. Additionally, a Chi-Square test was applied to examine the association between categorical variables, such as stunting status and participation in interventions, as well as to assess proportional changes pre- and post-intervention. These statistical analyses provide a comprehensive overview of the intervention's impact, with the most notable improvements observed in Denggen Village. Conducting a comparative analysis of the available literature is a crucial step to understanding the developments, methodological approaches, and outcomes achieved in a field of study [69].

#### 3.3.2 Machine learning model performance

The machine learning models were trained using pre-intervention baseline variables: five numeric features (age [months], weight [kg], height [cm], MUAC [cm], head circumference [cm]) and two encoded categorical variables (gender, mother's education level). The machine learning model was built using only numeric and coded categorical variables obtained at baseline, without including intervention variables, to ensure that predictions were based on data prior to treatment. The Gaussian Naïve Bayes and Decision Tree C4.5 algorithms were selected based on their proven performance and interpretability in health-related classification problems, particularly in stunting and nutritional status prediction as reported in previous studies [70]. Gaussian Naïve Bayes was chosen for its ability to handle small datasets and its robustness in cases with normally distributed continuous features. Decision Tree C4.5 was included due to its capability to model non-linear relationships, handle both numeric and categorical data, and provide transparent decision rules that are easy to interpret for public health practitioners [71]. This combination [72] allows us to compare a probabilistic generative model with a rule-based model, offering complementary perspectives for prediction and interpretation. This research uses the Gaussian Naive Bayes and Decision Tree C4.5 methods for the classification of stunting and non-stunting models. Gaussian Naive Bayes was chosen as the baseline model due to its simplicity and ability to handle data with a Gaussian distribution, while the Decision Tree Classifier was configured with the *class_weight* *=* *‘balanced’* parameter to internally address data imbalance. For model validation, a K-Fold Cross Validation technique of 5 folds was used with *shuffle* *=* *True* and *random_state* *=* *42* to ensure the randomized data remained consistent between trials. Performance evaluation was performed at each fold using accuracy metrics, classification report (including precision, recall, and f1-score), and confusion matrix. In addition to the use of *class_weight*, class imbalance was addressed through oversampling using the Synthetic Minority Over-sampling Technique (SMOTE) as a data preprocessing step [[73], [74], [75]]. SMOTE was applied exclusively to the training data after the split in each fold within the K-Fold cross-validation loop to avoid data leakage. This preprocessing method generates synthetic samples for the minority class to achieve a balanced class distribution in the training set, which can improve the classifier's ability to detect the minority class, as reported in other stunting prediction studies [76]. The results showed that model accuracy across all folds was consistently high, indicating that the baseline features were capable of effectively distinguishing between classes. Importantly, no features contained target information directly, and the balancing process was performed strictly on the training data without involving the test set.

Finally, to answer RQ3, baseline variables were modeled using machine learning algorithms, with performance results presented in Table 14 and Table 15.

**Table 14:** Comparison of Gaussian Naive Bayes and decision tree performance per class.

Model	Class	Precision	Recall	F1-score	Accuracy
Gaussian Naïve Bayes	0	1.0000	1.0000	1.0000	0.9971
	1	0.9800	1.0000	0.9900	
Macro avg	–	0.9900	1.0000	0.9950	
Decision Tree	0	1.0000	1.0000	1.0000	1.0000
	1	1.0000	1.0000	1.0000	
Macro avg	–	1.0000	1.0000	1.0000

**Table 15:** Confusion matrix for gaussian naïve bayes and decision tree using SMOTE.

Model			Predicted label	
			Not stunting	Stunting
Gaussian Naïve Bayes	True Label	Not Stunting	294	1
		Stunting	0	47
Decision Tree	True Label	Not Stunting	295	0
		Stunting	0	47

Table 14 shows very high performance in both classes. Gaussian Naive Bayes achieved perfect precision, recall, and F1-score (1.00) in class 0, but in class 1, precision decreased slightly to 0.98 with recall remaining at 1.00, resulting in an F1-score of 0.99 and an overall accuracy of 0.9971. The Decision Tree achieves perfect scores (1.00) on all metrics in both classes, with a total accuracy of 1.0000.

Table 15 shows the results using the Gaussian Naive Bayes method which is 0.9970 while the Decision Tree C4.5 method gets a result of 1.0000. This shows that the ideal method used in this case is Decision Tree. The division of data used as training and testing data is 70 versus 30. To ensure a fair and reproducible evaluation, the dataset was first split into 70 % training and 30 % testing portions, which is a commonly adopted ratio for small-to-medium datasets to provide sufficient samples for both model learning and generalization assessment. Within the training subset, a 5-fold cross-validation (K-Fold CV) with shuffle = True and random_state = 42 was applied to further validate the model stability and mitigate overfitting.

This two-stage validation design initial 70:30 split followed by internal cross-validation on the training set—was chosen to balance between model evaluation reliability and computational feasibility given the dataset size. The Decision Tree method is superior in terms of accuracy compared to Gaussian Naive Bayes, but not in computation time.

#### 3.3.3 Relationship between intervention variables and ML predictions

1) Multidisciplinary Approach

The multidisciplinary approach in this study involves a combination of health, religion and IT sciences, providing a holistic solution to the problem of stunting. The results show that each discipline plays an important and complementary role:

a) Health and Nutrition: Supplementary feeding and nutrition education are important interventions tailored to the needs of children at risk of stunting, ensuring that their nutritional needs are met.
b) Religion (Islamic values): Islamic values-based approaches, such as the importance of hygiene, responsibility in caring for children as a trust, and understanding the importance of health, increase family acceptance and compliance with the program.
c) Technology: Measuring habits and patterns in data to model new data with similar cases.

2) Trend Data

The effect of data trends in the study across multiple timeframes before and after the intervention. It can be used as a guide that the resulting data model is the determinants of change, including the location of the area far from the location of the Community Health Center, the condition of the infant's mother's lack of knowledge about healthy living, as well as the condition of the mother's health and age during pregnancy.

3) Anti-stunting Education

The Islamic values-based anti-stunting education approach proved effective in improving families' understanding and awareness of the importance of nutrition, hygiene and mental health. Results show that education tailored to the community's cultural beliefs and values increases participation as well as overall acceptance of the program. Some of the key outcomes of this approach include:

a) Increased Awareness and Knowledge: Education covering aspects of balanced nutrition, the importance of sanitation, and the impact of mental health on child development increased family knowledge.
b) Changes in Daily Habits: Through teaching about hygiene in Islam, families are more disciplined in maintaining a clean home environment and sanitation, which has a direct impact on reducing the risk of infectious diseases.
c) Spiritual Motivation: Understanding the importance of maintaining children's health as a mandate from Allah creates strong spiritual motivation, which makes families more committed to practices that support child development.
d) Community Empowerment: This education also integrates the role of community, where families support each other in practicing healthy habits, creating a strong social support network.

4) Machine Learning Model

The research also produced a machine learning-based prediction model that aims to identify and monitor children at risk of stunting. The model is based on data on nutrition, hygiene, mental health conditions, and other demographic factors, with several results such as Prediction Accuracy, Early detection system, and Long-term Prediction that predicts the risk of stunting in the future.

## 4 Discussion

Like other studies that have been conducted by researchers to predict diseases [23,[77], [78], [79], [80], [81], [82], [83]] in the health world using Machine Learning approaches [[84], [85], [86], [87], [88]] to other multidiscipline [10,26,[89], [90], [91], [92]]. This is the focus of our research this time, combining several multidisciplinary approaches to be able to make machine learning models as an application pattern in handling stunting disease [24,93,94]. As has been done in several recent studies using mixed methods [[95], [96], [97]], the interventions carried out on stunting have successfully achieved high accuracy.

We located data on pregnant women collected from January 2023 to January 2024 in the study area. Based on the data, there are several factors that also influence the risk of stunting in children, which calls for an approach that does not solely focus on the issue of child malnutrition, in line with the study by Sadler, K. et al. (2022) [24]. These are described as a history of disease or complications during pregnancy, diseases such as anemia and age that can affect the risk. This is in accordance with the results of research conducted by Abdullah, A et al. (2025) who found that informal interventions affect the prevalence of stunting in a region [98]. We try again to describe post-pregnancy data with the presence of several interventions as a comparison of the effect on this stunting case. The research team works with partners or local health services to collect data, carry out interventions to monitoring and evaluation that focuses on the community health center in Denggen village.

We obtained a comparison of stunting status by age between February 2024 (baseline) and August 2024 (endline) data. Based on age, there was little change. Likewise, with the results based on location, only Denggen village has a decrease, but it is not significant if the *p*-value is calculated. This is because the Denggen area is close to the coverage of puskesmas locations that are monitored by the system. Interestingly, Denggen Village was the only area showing a positive trend in reducing stunting prevalence, although the change was not statistically significant. Several contextual factors may explain this relative success. Denggen's proximity to the community health center allowed more intensive supervision, follow-up visits, and accurate monitoring. Stronger community participation, supported by local religious leaders, enhanced the acceptance of Islamic value–based education and encouraged mothers' active involvement in nutrition and hygiene sessions. The village also had slightly better socio-economic and infrastructure conditions, including higher maternal literacy and improved access to clean water and sanitation, which reinforced the intervention's behavioral components. Moreover, active engagement of health cadres and local authorities ensured adaptive management and timely responses to implementation challenges. These combined factors suggest that Denggen's improvement resulted from the synergy between accessibility, community engagement, and value-based education, rather than from the intervention design alone. Similarly, another study revealed that coverage by the system was associated with reducing the prevalence of child stunting [6]. The center for monitoring, measurement and evaluation is located at the agency, so the effectiveness of the number of respondents measured is limited due to demographic issues. However, these demographic and geographic issues were not investigated as instruments for the characteristics that were intervened. Some characteristics that were intervened with had significant results such as aspects of health, nutrition, and mental health.

Although *p*-values were reported to determine statistical significance, additional interpretation was enhanced by incorporating effect size and confidence intervals. The modest Cohen's d values in knowledge and attitude changes suggest meaningful practical impacts, even where statistical significance alone may not fully capture the intervention's influence. Moreover, confidence intervals around village-level changes highlight regional disparities. Some villages, such as East Denggen and Rakam, had statistically significant increases in stunting, possibly due to contextual challenges, as reflected in the moderate Cramer's V values. These insights emphasize the importance of looking beyond p-values to assess the robustness and policy relevance of stunting interventions.

In addition to statistical outcomes, the education component of this intervention showed notable improvements in knowledge and attitudes of mothers toward stunting prevention. Knowledge increased from 76.9 % to 92.3 % after the intervention, with a moderate-to-large effect size (Cohen's d = 0.68), while attitudes improved from 61.5 % to 73.1 % (Cohen's d = 0.51). However, behavior change remained stagnant at 61.5 %, suggesting that while the program effectively enhanced awareness and motivation, translating these gains into daily practices requires longer-term support. This finding aligns with behavioral change theory, which emphasizes that knowledge and attitudes are precursors but not guarantees of sustained practice. Environmental constraints, economic limitations, and childcare arrangements (e.g., reliance on caregivers when parents work abroad) likely restricted the translation of educational gains into consistent behavioral outcomes. Notably, aspects of hygiene and nutritional fulfillment showed statistically significant relationships with improved stunting outcomes, confirming that value-based education can reinforce practical behaviors when coupled with supportive conditions. Conversely, the infant health aspect showed weaker results, possibly due to the short monitoring period and external constraints on vaccination and growth monitoring.

Modeling results using machine learning with Gaussian Naive Bayes and Decision Tree C4.5 are very effective in handling stunting cases [99]. The data imbalance problem in this study was successfully addressed through the application of the SMOTE method, which significantly improved the model's ability to generalize and classify minority cases accurately. Like other studies that employed SMOTE-based balancing techniques [22], the integration of this method proved to be a decisive factor in enhancing predictive performance across imbalanced health datasets. As demonstrated in previous research conducted in sub-Saharan Africa, where advanced algorithms such as Random Forest, XGBoost, and Deep Neural Networks were evaluated, the implementation of SMOTE and class weighting strategies substantially improved model performance—yielding excellent accuracy, precision, recall, F1-score, and AUC-ROC results. Their results demonstrated near-perfect prediction rates, indicating that balancing techniques like SMOTE are critical for optimizing classification outcomes and ensuring the robustness of stunting prediction models.

This finding aligns with the current study, suggesting that even relatively simple algorithms such as Naive Bayes and Decision Tree can achieve reliable performance improvements when combined with SMOTE-based balancing. Therefore, future work should continue refining the integration of SMOTE with various classifiers to ensure stability and reproducibility across different socioeconomic and geographical settings. Furthermore, while the current study emphasizes short-term post-intervention outcomes, long-term follow-up monitoring is planned to evaluate the sustainability of intervention effects by incorporating socioeconomic and geographical indicators. This approach is expected to strengthen the external validity of the findings and support the scalability of data-driven stunting prevention models across diverse regions.

Although the Decision Tree and Naïve Bayes models achieved near-perfect accuracy, this outcome must be interpreted with caution. The inclusion of raw age and height as predictors may inadvertently replicate the WHO Height-for-Age z-score (HAZ) definition of stunting, thereby inflating performance metrics. In such cases, the models may not reflect true predictive novelty but rather reproduce established classification criteria. The primary rationale for including machine learning in this study was to explore its potential as a decision-support tool that can be expanded to integrate non-anthropometric variables, such as socioeconomic status, maternal literacy, hygiene practices, and participation in Islamic value-based interventions, which go beyond WHO's standard definition. This extension could provide meaningful predictive insights, especially in settings where complete anthropometric data may be unavailable

One of the main limitations lies in the potential bias in the data collection process, especially since the data was obtained from a specific study area that has distinctive social, economic and cultural characteristics. This may affect the model's results in identifying stunting cases, so its application in other regions with different contexts needs to be done with caution. Although the model performed very well in the validation process, this does not necessarily reflect the same performance when applied outside the data sample used in this study. Machine learning models are also highly dependent on the quality and completeness of the features used. Therefore, if the input variables are not fully available in other regions, the effectiveness of the model may decrease. In the future, the generalizability of the model can be improved by expanding the coverage of data from different regions and involving external evaluation approaches to test the robustness and portability of the model.

In addition, this study is limited by the relatively short duration of the intervention, which lasted only six months. This time frame may not have been sufficient to observe long-term behavioral and nutritional changes or their cumulative effects on child growth. Furthermore, the research was geographically constrained to several villages with specific socio-economic and cultural characteristics, which may introduce contextual bias and limit the generalizability of the findings to other regions. Future studies are therefore encouraged to employ longitudinal designs and include more diverse regional samples to enhance external validity and ensure that the intervention's outcomes are sustainable across different contexts.

One major limitation of this study is that the very high accuracy of the machine learning models likely stems from the use of age and height as predictors, which are directly tied to WHO's definition of stunting. As a result, the models may simply replicate the existing classification rather than provide novel predictive power. To address this, we performed a sensitivity analysis excluding these variables, which resulted in a substantial drop in accuracy, confirming their dominant role. This limitation highlights the need for future studies to incorporate broader variables—such as socioeconomic, environmental, and behavioral factors—to develop more robust and generalizable prediction models.

The interventions undertaken in this study such as the use of educational models have been applied in several previous studies and found to be effective in their application [100]. The concept of religion is one of the intervention options carried out in this study by using an educational model that comes from the Qur'an and Hadith. Several determinants of stunting such as the environment, habits to eat protein foods. For example, protein food sources that are allowed (halal) and not (haram) in Islamic teachings also need to be considered and contained in the Qur'an and Hadith [101]. Because the guidelines in Islam encompass all aspects, including a healthy lifestyle as practiced by the Muhammad SAW, this aligns with findings from other studies [102]. Similarly, the machine learning approach has also been widely used in recent years to predict stunting [103].

Despite comprehensive interventions, it is important to note that some significant determinants of stunting still show high prevalence in the study sites, such as family income level, maternal education, and age of mother and child. These factors have long-term effects that do not necessarily change through short-term interventions [41]. These factors have long-term effects that do not necessarily change through short-term interventions.

On the other hand, the study also noted positive behavioral changes in participants in response to the intervention. In the motivation aspect, there was an increase in consistency in parents' commitment to childcare. In terms of hygiene, respondents demonstrated the habit of washing hands, using clean water, and utilizing sanitation facilities such as toilets. In the nutritional fulfillment aspect, there was an increase in breastfeeding, complementary feeding (MP-ASI), and the completeness of child immunization. In terms of mental health, there was awareness of sleep disorders and anxiety among mothers, indicating an increased understanding of the importance of psychological health in parenting. Meanwhile, in infant health, there were improvements in monitoring children's physical growth, intellectual and emotional development.

However, while these positive behavioral changes have been recorded, they have not directly resulted in a significant reduction in stunting prevalence. This suggests that addressing stunting requires a more systemic and sustainable approach, which not only focuses on individual behavior change, but also addresses broader structural factors. This finding is in line with a study by Christian, Parul et al. (2020) [104] which evaluated the impact of a comprehensive nutrition program in rural Malawi. The study showed that despite improvements in complementary feeding practices and handwashing habits, the prevalence of stunting did not decrease significantly. This confirms that behavioral change alone is not enough if it is not accompanied by improvements in socio-economic aspects and access to basic services.

Furthermore, stunting interventions not only impact health aspects, but also have long-term socio-economic implications. Stunted children are at risk of cognitive delays that impact educational attainment and future economic productivity. Thus, the success of this intervention has the potential to improve the quality of human resources and break the cycle of intergenerational poverty, especially in areas with limited access to health and education services.

This study has the strength of combining machine learning models proven to be robust through cross-validation, holistic interventions. These range from nutrition, hygiene, motivation, fulfillment, mental health to infant care that are also aligned with Qur'anic and Hadith values. This increases community acceptance, supported by pre-test and post-test designs that provide direct empirical evidence of changes in nutritional status and behavior.

Although the Decision Tree and Naïve Bayes models achieved near-perfect accuracy, this outcome must be interpreted with caution. The inclusion of age and height as input variables may replicate the WHO HAZ criteria, potentially inflating accuracy. A sensitivity test excluding these variables resulted in a notable decline in model performance, confirming their dominant role. Future models should therefore prioritize non-anthropometric variables—such as socioeconomic, environmental, and behavioral factors—to enhance generalizability and ensure true predictive validity.

For the intervention to have a long-term impact, the sustainability of the program after the study period is crucial. One of the main strategies proposed is to involve local government, posyandu cadres, and community leaders in the implementation and monitoring process. This aims to create a sense of ownership and collective responsibility for the success of the program. In addition, the intervention's religious values-based approach allows for integration into routine socio-religious activities, such as khutbah, thus strengthening sustainability through cultural and spiritual approaches. For scaling up to other areas, it is important to adapt the intervention design to the local context, as well as establish cross-sectoral cooperation between the health office, education office, and civil society organizations. With good documentation, cadre training based on standardized modules, and policy support, this intervention has high potential to be widely and sustainably implemented as part of the national stunting strategy. At the same time, it considers the long-term socio-economic impact in breaking the cycle of poverty and produces a framework that is ready to be used as a policy guide with high potential for scale and portability to other areas.

## 5 Conclusions

This study concludes that a multidisciplinary approach, trend data analysis, Islamic values-based education, and the application of machine learning models are potentially comprehensive solutions in addressing the issue of stunting in Indonesia. The distribution data included 3416 children studied, with the gestational age of the mothers ranging between 17 and 37 months. In addition, trend data analysis revealed key risk patterns associated with stunting, such as maternal conditions during pregnancy (age and comorbidities), demographic and geographic factors, nutritional intake, parental education, and socioeconomic status. The data shows that 41.5 % of pregnant women were at high risk of giving birth to a stunted child.

The analysis based on age and location revealed that only Denggen Village experienced a non-significant yet positive decrease in stunting cases (*p* = 0.176; z = 1.35), while other villages showed no statistically significant changes. Interviews with research partners indicated that one of the contributing factors was the presence of a Community Health Center (Puskesmas) in the village, which facilitated better monitoring and evaluation of the respondents. Testing was also conducted through descriptive analysis covering understanding of stunting, stunting prevention attitudes, and behavioral aspects, which showed an improvement of 71.3 %. Demographic characteristics also received particular attention: 76 % of respondents were over 25 years old; 34.6 % had completed junior or senior high school; 42.3 % had a monthly income ranging from IDR 250,000–500,000; 76.9 % were housewives; 100 % were married; 38.5 % had experienced pregnancy once; 92.3 % had a history of childbirth; and 84.6 % had no history of anemia.

*P*-value calculations based on interventions - covering aspects such as Motivation, Hygiene, Nutritional Fulfillment, Mental Health, and Infant Health - showed that the Hygiene Aspect, Nutritional Fulfillment Aspect, and certain parts of the Mental Health Aspect had statistically significant results. Furthermore, the study successfully developed machine learning models with very high accuracy: 100 % using the Decision Tree algorithm and 99.7 % using the Gaussian Naive Bayes algorithm, to predict whether a child would experience stunting or not. This study further reinforced its findings with statistical rigor by presenting effect sizes and confidence intervals, which enhanced the understanding of the intervention's magnitude and variability. While Denggen showed a non-significant but positive trend, other villages demonstrated mixed results, highlighting the contextual variability and the need for further investigation before broad conclusions can be drawn. These results affirm the necessity of nuanced, location-sensitive strategies in anti-stunting interventions.

Future studies are encouraged to validate the proposed models using longitudinal and multi-regional datasets to ensure broader generalizability. Practical implementation should focus on developing a digital monitoring and decision-support system that integrates community health data with machine learning analytics for real-time stunting risk prediction. Moreover, collaboration between health institutions, educational organizations, and religious leaders is recommended to strengthen community engagement and promote sustainable behavioral changes based on Islamic values. Expanding these interventions through policy integration and cross-sectoral partnerships will enhance the national capacity to prevent and reduce stunting effectively across diverse regions in Indonesia. Overall, this approach indicates potential effectiveness within the specific demographic and regional context of this study and is consistent with similar community-based intervention studies, while further validation remains essential for broader applicability.

## Statement on generative AI and AI-assisted technologies in the writing process

During the preparation of this work the author(s) used ChatGPT (OpenAI) and DeepL Translator to support language editing, translate comments from reviewers, and discuss sentence structure of the ideas provided. After using this tool/service, the author(s) reviewed and edited the content as needed and take(s) full responsibility for the content of the publication.

## CRediT authorship contribution statement

**Mhd. Lailan Arqam:** Writing – review & editing, Supervision, Project administration, Funding acquisition. **Asno Azzawagama Firdaus:** Writing – review & editing, Writing – original draft, Supervision, Software, Methodology, Conceptualization. **Ahmad Muslih Atmojo:** Writing – review & editing, Conceptualization. **Ginanjar Zukhruf Saputri:** Writing – review & editing, Validation, Methodology, Conceptualization. **Furizal:** Validation, Formal analysis. **Palahuddin:** Resources, Data curation. **Retno Sirnopati:** Validation, Data curation.

## Funding statement

This research was supported by the 10.13039/501100002701Ministrya of Education, Culture, Research and Technology of the Republic of Indonesia based on the Decree of the Budget User Authority Number 0459/E5/PG.02.00/2024 with the Institution Master contract number between DRTPM and LLDIKTI V: 107/E5/PG.02.00.PL/2024 dated June 11, 2024.

## Declaration of competing interest

None.
